# Paleozoic diversification of terrestrial chitin-degrading bacterial lineages

**DOI:** 10.1186/s12862-019-1357-8

**Published:** 2019-01-28

**Authors:** Danielle S. Gruen, Joanna M. Wolfe, Gregory P. Fournier

**Affiliations:** 1 Department of Earth, Atmospheric and Planetary Sciences, Massachusetts Institute of Technology, Cambridge, MA 02139 USA; 2 Department of Marine Chemistry and Geochemistry, Woods Hole Oceanographic Institution, Woods Hole, MA 02543 USA

**Keywords:** Horizontal gene transfer, Chitinase, Chitin, Bacteria, Fungi, Arthropods

## Abstract

**Background:**

Establishing the divergence times of groups of organisms is a major goal of evolutionary biology. This is especially challenging for microbial lineages due to the near-absence of preserved physical evidence (diagnostic body fossils or geochemical biomarkers). Horizontal gene transfer (HGT) can serve as a temporal scaffold between microbial groups and other fossil-calibrated clades, potentially improving these estimates. Specifically, HGT to or from organisms with fossil-calibrated age estimates can propagate these constraints to additional groups that lack fossils. While HGT is common between lineages, only a small subset of HGT events are potentially informative for dating microbial groups.

**Results:**

Constrained by published fossil-calibrated studies of fungal evolution, molecular clock analyses show that multiple clades of Bacteria likely acquired chitinase homologs via HGT during the very late Neoproterozoic into the early Paleozoic. These results also show that, following these HGT events, recipient terrestrial bacterial clades likely diversified ~ 300–500 million years ago, consistent with established timescales of arthropod and plant terrestrialization.

**Conclusions:**

We conclude that these age estimates are broadly consistent with the dispersal of chitinase genes throughout the microbial world in direct response to the evolution and ecological expansion of detrital-chitin producing groups. The convergence of multiple lines of evidence demonstrates the utility of HGT-based dating methods in microbial evolution. The pattern of inheritance of chitinase genes in multiple terrestrial bacterial lineages via HGT processes suggests that these genes, and possibly other genes encoding substrate-specific enzymes, can serve as a “standard candle” for dating microbial lineages across the Tree of Life.

**Electronic supplementary material:**

The online version of this article (10.1186/s12862-019-1357-8) contains supplementary material, which is available to authorized users.

## Background

Dating when new metabolisms evolved and when major clades of Bacteria arose, particularly on the order of hundreds of millions of years, remains a key challenge in biology [1]. Despite progress in understanding the molecular record of extant bacterial genomes, the timing of the evolution of major clades of Bacteria is especially problematic to resolve due to complex gene histories and a lack of clear phenotypic traits that can be correlated with a diagnostic fossil record [2]. In the near-absence of physical (geochemical or fossil) records of microbial evolution, it is difficult to determine and date the evolutionary history of bacterial lineages [3].

Leveraging the information contained in horizontal gene transfer (HGT) events can substantially improve estimates of the timing of events within microbial evolution [4–9]. Vertical inheritance passes genetic information from parent to offspring, but HGT passes genetic information between organismal lineages, across all degrees of evolutionary distance. This can be particularly useful for molecular clock dating, as HGTs establish cross-cutting relationships between lineages and serve as a “temporal scaffold” upon which fossil calibrations or other date information from even distantly related taxa may be placed [5, 8, 10]. While HGT is a major process in microbial evolution [7, 11], HGT events between microbes and eukaryotes with a fossil record are less frequently identified [12]. Furthermore, the donor-recipient relationships are often difficult to infer for many gene histories due to multiple HGT events and gene losses or the lack of a strong phylogenetic signal [13]. The function of a gene is not necessarily relevant to its utility in propagating time constraints (e.g. [4]); however, in some cases, this gene function may be additionally informative, and provide independent support for age estimates. This is the case, for example, if the protein encoded by the transferred gene is specific for a substrate that can, itself, be temporally constrained. Given all of these criteria, a very small number of HGT events may be especially valuable for dating microbial lineages; these “index transfers” [9] can be even more valuable if multiple HGT recipients are present, closely correlating the ages of the recipients in time, a “standard candle” (a term used in astronomy to describe an object with known luminosity used to infer the cosmic distances to other objects of interest) [14].

### Environmental distribution of chitin

Chitin is one of the most abundant structural polysaccharides in nature [15, 16], and chitin degradation by chitinase enzymes is a critical process in the biogeochemical cycling of carbon and nitrogen in terrestrial and aquatic ecosystems [15]. There are two dominant biogenic sources of chitin: arthropods [16] and fungi [16]. Chitin may therefore have increased in abundance in terrestrial systems following the terrestrialization of arthropods, sometime after the Cambrian [17]. In modern aquatic systems, arthropods are the dominant chitin-producing organisms. While there is a great deal of uncertainty in these estimates, the chitin sourced from arthropods is roughly 2.8 × 10^7^ Mg yr^− 1^ in freshwater ecosystems and 1.3 × 10^9^ Mg yr^− 1^ in marine ecosystems [18]. The majority of chitin in terrestrial ecosystems is produced by fungi [19] largely due to their contribution of biomass to the soil environment [20]. While global estimates for the contribution of arthropod biomass, and thus chitin, to the environment over time are lacking, arthropods nonetheless make up the largest pool of animal biomass today [21].

### Chitin production and the evolution of Fungi

The evolution of chitin producers is anchored to the fossil record through diagnostic morphological characters [22–26]. In the case of Fungi, Cryptomycota form the most deeply branching fungal clade, and contain the most deeply branching chitinous Fungi (e.g., *Rozella)* [24, 25]. Fossil-calibrated molecular clock studies generally agree that early Fungi diverged around 1145–738 million years ago (Ma) [27]. Fossil and molecular clock evidence also indicates that divergence of Ascomycota and Basidiomycota within the major fungal group Dikarya occurred around 830–518 Ma [24] with a fossil minimum around 405 Ma [23, 28, 29]. Posterior age estimates from molecular clock studies suggest that crown Ascomycota diversified 715–408 Ma [30] and crown Basidiomycota diversified 655–400 Ma [28]. Therefore, studies of fungal evolution can inform the timing of chitinase gene evolution.

Based on fossil and molecular clock dating methods, marine crown-group euarthropods appeared around 521–514 Ma, shortly after the start of the Cambrian, and radiated into the lower and middle Cambrian [29, 31]. Molecular clock and fossil evidence suggests that terrestrialization of major arthropod groups occurred from the Cambrian into the Silurian [32]. ﻿The oldest terrestrial myriapod body fossil (the oldest undisputedly terrestrial animal) is the 416 Ma *Crussolum sp.* [29]. However, the radiation of terrestrial arthropods (including insects) likely continued into the Devonian [17, 33, 34].

### The evolution of chitinase gene families

Chitinases are proteins that catalyze the breakdown of glycosidic linkages in polymers of chitin [16]. Chitinases are a type of glycoside hydrolase (GH) specific to chitin [16, 35]. There are two main families of chitinases: glycoside hydrolase family 18 (GH18) and glycoside hydrolase 19 (GH19) [16]. GH18 chitinases are distributed across the three domains of life [16, 36], whereas GH19 chitinases are restricted to mostly plants and are rarely associated with bacteria [36]. In one well-studied bacterial model, *Streptomyces*, there were ten genes associated with the GH18 family of chitinases (homologs chiA-E, and H- L) and two genes associated with GH19 (chiF, G) [37]. It has been suggested that some of these genes may have evolved under selective pressures related to the host environment or to the presence and proximity to other organisms, which may have even precipitated HGT events [37–39]. Myxobacterial chitinases have been hypothesized to have evolved via HGT [40], and other bacterial lineages within Actinobacteria are hypothesized to have co-opted a fungal chitinase for self-defense [37]. Because of the specific associations between substrate and gene, it stands to reason that there may be an evolutionary link between the major producers of environmental chitin (fungi and arthropods) and chitin-degrading genes in bacteria. It has been shown that some bacterial chitin degradation systems are even adapted to the environments (aquatic vs. terrestrial) and most abundant chitin producers (exoskeletons of crustaceans vs. fungal cell walls) that they encounter [15]. Nonetheless, it remains to be tested whether chitinase genes also reflect widespread environmental adaptations over geological time.

It has been shown that chitinases may retain a molecular record of evolutionary events hundreds of millions of years ago [41]. While some of the phylogenetic distribution of these genes may indicate a pattern of vertical inheritance, other chitinase genes may have evolved via horizontal gene transfer [37]. For these reasons, and the criteria described above, chitinase genes are an attractive potential source of temporal information for microbial evolution. Therefore, we sought to test the hypothesis that specific bacterial chitinases evolved via HGT, and if so, if these HGT events could be leveraged to propagate known fossil calibrations between donor and recipient lineages. Bacterial chitinases are especially useful because they metabolize chitin, a specific biopolymer only produced in abundance by arthropods and fungi, two groups with fossil records, and thus likely age estimates, much more precise than those of most microbial groups. Previous work has also suggested that some chitinases are distributed between the domains of life via HGT, for example, postulating that some chitinase genes were transferred from plants to Actinobacteria and then to arthropods [42]. However, the evolutionary history of the many disparate chitinase gene families in microbes has not been fully investigated.

### Bayesian molecular dating

Fossil-calibrated molecular clock models are applied to estimate divergence times of organisms (e.g. [3, 43]). Many divergence time analysis parameters have only been recently developed, and few have been applied to microbes with divergence time estimates that span geologic time or have undergone rampant horizontal gene transfer events (e.g. [8, 44]). For a more detailed review of these parameters and challenges see, for example, [43, 45–53]. The issues inherent to assessing microbial evolution present a challenge for this work, but also an opportunity to explicitly test these model parameters and assumptions in order to determine those that are valid for this specific set of evolutionary conditions.

Molecular clock dating is based on a Bayesian framework, reviewed in greater detail by others [51, 52, 54]. There are a few major components used to determine posterior probabilities or date distributions such as data selection, calibrations, the molecular clock model, the tree process prior, and the rate distribution model. The sequence data assessed in this work are the chitinase genes present in bacterial and eukaryotic lineages. Tree process priors include birth-death and uniform. Rate distribution models include lognormal autocorrelated and uncorrelated gamma.

We tested the uniform prior and the birth-death tree process priors. The uniform prior considers every possible topology to be equal and favors divergences that are evenly spaced across the tree from the root to tip [55, 56]. The birth-death model is defined by speciation (“birth”) and extinction (“death”). In contrast to the uniform prior, this tree process ascribes more weight to tree topologies with certain branching patterns [57]. The birth-death process generally biases the model such that deeper branches are longer and the more shallow branches are shorter, because it is assumed the “older” lineages more often end in extinction [52]. Biases such as this can have large effects on the posterior age estimates and inappropriate model selection can result in less precise dates.

All models in this study assume a relaxed molecular clock model for a prior on the branch rate. However, two relaxed clock models for the branch rates are assessed: autocorrelated and uncorrelated. Uncorrelated clocks make no assumption that branches next to each other on the tree should share similar rates. In other words, the rate on each branch of the tree is independent. Conversely, autocorrelated clocks assume that more closely related branches on the tree should also have more similar rates [46, 56, 58, 59]. The assumption that neighboring branches should share more similar rates makes sense when we consider that the evolution of genetic information between related lineages is often affected by many of the same processes that affect the rates of evolution (e.g. environment, population) [52]. Biological events such as horizontal gene transfer may invalidate model assumptions, but the mechanisms of rate variation and quantifying the relative importance of various biological events are still debated [1]. Choosing between these models is a matter of ongoing debate in the field, and is often dependent on the data [52, 56, 60]. Thus, we detail the effects of model selection in our analyses.

The primary objective of this work is to test whether fossil-calibrated age estimates within fungi can be propagated to bacterial lineages through the use of HGT events between these lineages under different model assumptions. Secondarily, we seek to understand possible ecological implications of the evolution of chitinases in fungi and bacteria. If bacterial chitinase genes were acquired in response to environmental chitin availability, then arthropod evolutionary history provides a prediction for the timing of these events within bacterial lineages. We hypothesize that terrestrial bacterial chitinases diversified from the Cambrian into the Devonian following the distribution of environmental chitin. We independently date chitinase evolution in microbial lineages by first testing and then applying molecular clock models to chitinase gene trees, constrained by fungal date calibrations tethered via HGT. We show that certain model parameters seem to outperform others. Moreover, our posterior date distributions for bacterial lineages support the utility of HGT-propagated fossil calibrations in accurately estimating the ages of microbial lineages as an avenue for future work.

## Methods

### Taxon sampling

We queried The National Center for Biotechnology Information (NCBI) nonredundant (nr) database using the protein Basic Local Alignment Search Tool (BLASTp) for sequences homologous to the *Myxococcus fulvus* ChiD protein (WP_046715376.1). Complete protein sequences of the top 5000 hits from NCBI were downloaded (E-value < 10^− 5^). Sequences were subsampled from this list to include to include a single representative of each species as annotated in NCBI, to avoid an overabundance of terminal taxa representing multiple strain isolates of the same species. We further used BLASTp to more exhaustively identify potential homologs within Fungi, repeating this method for specific searches within Ascomycota, Basidiomycota, and more deeply-rooting Fungi (e.g. ﻿Blastocladiomycota, ﻿Chytridiomycota, ﻿Zoopagomycota, ﻿and Mucorales).

### Sequences and alignments

Sequences were aligned using the program MUSCLE [61]. Poorly aligning regions that contained misaligned gaps in the deeply rooting fungi were identified via manual inspection and removed using Jalview [62]. The resulting alignment was then manually edited to correct obvious misalignments in generally well-aligned regions adjacent to indels in *Fonsecaea multimorphosa* and *Phialophora americana* (sites 2390–2470). We also removed the misaligned C-terminal region from *Phelbia centrifuga*, and the misaligned C-terminal regions from *Rhizopus*, *Mucor*, *Synchephalastrum*, *Absidia*, and *Lictheimia* (sites 2393 onward). Both datasets (before and after trimming) are publicly available [63].

A profile alignment of bacterial and fungal sequences was made [61]. This revealed a highly conserved alignment region shared across bacterial and fungal sequences (sites 1844–2470) and another well-aligned N-terminal region conserved across Bacteria, but absent or poorly aligned in Fungi. In order to maximize the sequence information used for phylogenetic reconstruction and molecular clocks without introducing misalignments between bacterial and fungal sequences, a composite alignment was generated. This involved concatenating the conserved region for both Fungi and Bacteria with the N-terminal region aligned for just Bacteria. From this alignment, a single gene tree was generated for determining the relationship between Fungi and Bacteria, and for maximally resolving splits within the bacterial tree.

### Phylogenetic analyses

#### Gene tree

The gene trees were inferred using RaxML v1.8.9 using the PROTGAMMALGF substitution model [64] as fit by PROTTEST [65], and 100 bootstrap replicates. The resulting tree showed relationships between fungal taxa that are congruent with published phylogenies [22–26]. We rooted the gene tree on the branch leading to *Rozella*, which is considered to be either part of a sister group to the most deeply-rooting fungal clades, or a member of Chytridomycota, one of the most deeply-rooting Fungi [24]. This root resulted in bacterial chitinases as a clade diverging within crown Fungi, polarizing the origin of the bacterial homologs as originating via an HGT from a fungal donor. A consensus tree was generated from Bayesian Inference using PhyloBayes v.3.3 (CAT20 set of substitution models [60], effective size > 50, and variable discrepancies < 0.30).

#### Divergence time estimation

Divergence times were estimated using PhyloBayes v3.3 under the CAT20 set of substitution models [60]. Divergence time estimates were generated under several sets of model priors. Specific model parameters are described in Tables 1 and 2. After chain convergence (effective size > 50, variable discrepancies < 0.30), trees and posterior probability support values were generated from completed chains after the initial 20% of sampled generations were discarded as burn-in.

**Table 1:** PhyloBayes model parameters tested in this study. For each model, a +/− indicates the presence or absence of a condition. Sequence data were used to generate posterior probability distributions for all models, which were also tested under the prior by removing sequence data (−prior flag in PhyloBayes as indicated by a "p"). BD refers to birth-death. LN stands for lognormal autocorrelated. UGAM stands for uncorrelated gamma multipliers. The AB split refers to the split between Ascomycota and Basidiomycota. Fossil refers to the fossil minimum referenced in the Calibration Table (Table 2)

	Prior		Relaxed Clock Model		Calibrations				
Model	Uniform	BD	LN	UGAM	Root	AB split	A crown, B crown	Fossil	-prior
1	+	–	+	–	+	+	–	–	–
2	+	–	+	–	+	+	+	–	–
3	+	–	+	–	+	–	–	+	–
4	+	–	+	–	+	–	+	+	–
1p	+	–	+	–	+	+	–	–	+
2p	+	–	+	–	+	+	+	–	+
3p	+	–	+	–	+	–	+	+	+
4p	+	–	+	–	+	–	–	+	+
5	+	–	–	+	+	+	–	–	–
6	+	**–**	–	+	+	+	+	–	–
7	+	–	–	+	+	–	+	+	–
8	+	–	–	+	+	–	–	+	–
5p	+	–	–	+	+	+	–	–	+
6p	+	–	–	+	+	+	+	–	+
7p	+	–	–	+	+	–	+	+	+
8p	+	–	–	+	+	–	–	+	+
9	–	+	–	+	+	+	–	–	–
10	–	+	–	+	+	+	+	–	–
11	–	+	–	+	+	–	+	+	–
12	–	+	–	+	+	–	–	+	–
9p	–	+	–	+	+	+	–	–	+
10p	–	+	–	+	+	+	+	–	+
11p	–	+	–	+	+	–	+	+	+
12p	–	+	–	+	+	–	–	+	+
13	–	+	+	–	+	+	–	–	–
14	–	+	+	–	+	+	+	–	–
15	–	+	+	–	+	–	+	+	–
16	–	+	+	–	+	–	–	+	–
13p	–	+	+	–	+	+	–	–	+
14p	–	+	+	–	+	+	+	–	+
15p	–	+	+	–	+	–	+	+	+
16p	–	+	+	–	+	–	–	+	+

**Table 2:** Calibrations used in molecular clock models. All calibrations listed in Ma. Taxon 1 and Taxon 2 refer to the taxa used in PhyloBayes commands

Node	Taxon 1	Taxon 2	Calibration	Reference
Root	RozeAll205	Paeniba168	1145–738	Sharpe et al., 2015
AB Split	SerpLac210	TricVir244	830–518	Floudas et al., 2012
Ascomycota Crown	RoseNec204	MetaBru136	715–408	Prieto et al., 2013
Basidiomycota Crown	HessVes103	AgarBisp08	655–400	Floudas et al., 2012
Fossil minimum on AB Split	SerpLac210	TricVir244	830–405	Berbee and Taylor, 2010; Floudas et al., 2012

#### Date constraints

Secondary calibrations were applied to the divergence times of major fungal groups within the gene tree. For all analyses, we applied a root prior and one internal date constraint to the split of Ascomycota and Basidiomycota consistent with reported molecular clock and fossil evidence within Fungi [22–30, 66]. In order to avoid false precision, uniform priors were used in both cases, 1145–739 Ma for the fungal root [27] and 830–518 Ma for the Ascomycota-Basidiomycota split [28]. We also tested the addition of secondary calibrations on the nodes leading to the Ascomycota (715–408 Ma) and Basidiomycota (655–400 Ma) clades [28, 30]. Finally we tested the application of a primary fossil minimum calibration on the split of Ascomycota and Basidiomycota (830–405 Ma) [23, 28, 29]. All calibration structures are listed in Table 2.

## Results

### Phylogeny of ChiD and ChiC homologs

**Fig. 1:**
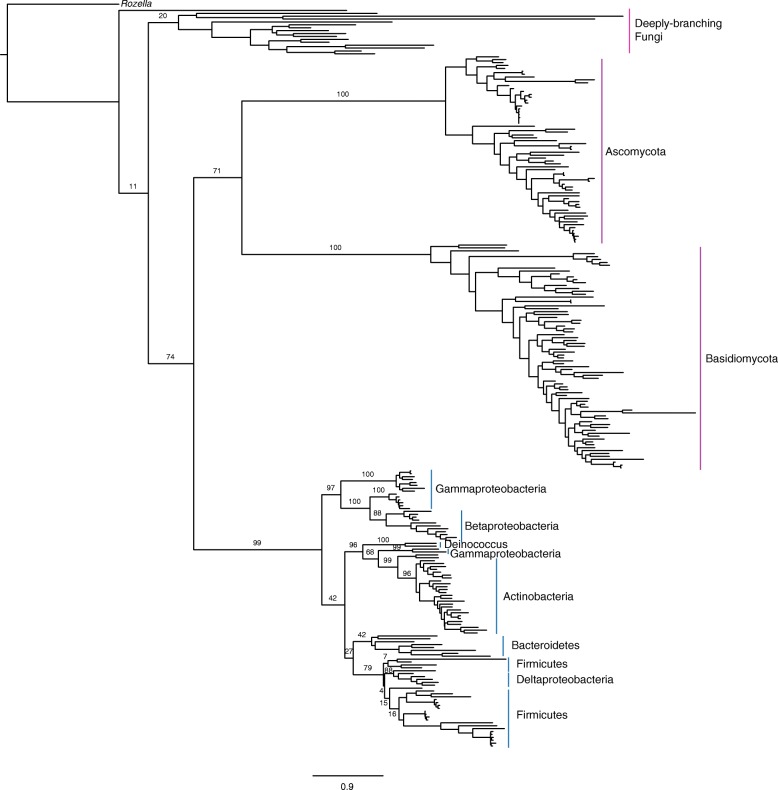
Chitinase Gene Tree. Maximum likelihood gene tree (RaxML) illustrating the relationship between fungal and bacterial taxa. Support values for within-family bipartitions were omitted for clarity, and can be accessed in Additional file 5: Figure S3

Figure 1 illustrates the relationships between sequences in this study (Additional file 1: Table S1) as a maximum-likelihood gene tree generated with RAxML. The tree is rooted with the most deeply-branching fungal taxon, *Rozella* (Cryptomycota). The group of deeply-rooting Fungi include members of Cryptomycota, Blastocladiomycota, Chytridiomycota, Blastocladiomycota, Chytridiomycota, Mucormycotina, and Zoopagomycota (in order of branching from the root), are generally consistent with recent results of phylogenomic analyses of the divergence of basal Fungi [67]. Bootstrap supports are low for many bipartitions within this deeply-rooting group. Support for the bipartitions placing bacterial sequences within Fungi are higher (74, 71). Support for the monophyly of Ascomycota and Basidiomycota is high (100). Support is also high for the monophyly of bacterial sequences (99). While the deeper branches in the fungal tree have weak bootstrap support, the relatively short branches relating these groups and the lack of any calibrations sensitive to their specific crown-group topology suggest the observed phylogenetic uncertainty has little impact on divergence times for more distal clades within the tree.

Within Bacteria are the generally well-supported and often monophyletic bacterial clades including groups within Betaproteobacteria (Burkholderiales, Chromobacteriaceae), Deinococcus, Actinobacteria, Bacteroidetes (Cytophagia, Flavobacteriacea, Chitinophagia), Firmicutes (Bacillales), and Deltaproteobacteria (Myxococcales). Gammaproteobacteria are polyphyletic, including Vibrionales, Xanthomonadales, and one Gammaproteobacteria taxon in Actinobacteria (*Cellvibrio*, WP_049631752.1), a cellulolytic bacterium in the order Pseudomonadales [68], suggesting multiple independent acquisitions of ChiD. Actinobacteria (bootstrap support 99%), Bacteroidetes (bootstrap support 42%), Firmicutes (bootstrap support 79%), and Deltaproteobacteria (bootstrap support 88%) are also monophyletic. Deltaproteobacteria sit on a reticulating branch within Firmicutes. The tree generated with PhyloBayes recovered a similar topology, further supporting the placement of the root within this group of deeply branching fungi (Additional file 2: Figure S1). Additional annotations for node numbers and clades are included in Additional file 3: Figure S2.

### Divergence time estimates of bacterial chitinases

Divergence time estimates were tested under several models, with the impacts of taxon sampling (inclusion or exclusion of bacterial sequences), tree priors (uniform vs. birth-death), and relaxed clock models (autocorrelated lognormal vs. uncorrelated gamma rate distributions) subsequently evaluated. Our preferred model is uncorrelated gamma distribution under a uniform prior with calibrations on the root (1145–738), Dikarya (830–518) and crown Ascomycota (715–408) and crown Basidiomycota (655–400).

Few published age estimates exist for the bacterial clades present in our tree. For example, the only other published divergence time estimate for Vibrionales (the last common ancestor of *Vibrio* and *Photobacterium*) was an uncalibrated RelTime clock built by using 16S rRNA and protein datasets [69]. The result for this clade was 124 Ma. Based on the chitinase HGT from a time-calibrated Fungi tree with a uniform prior and uncorrelated gamma clock model, the posterior age estimate for crown-group Vibrionales is ~ 188 Ma with an uncertainty spanning ~ 278–113 Ma.

**Fig. 2:**
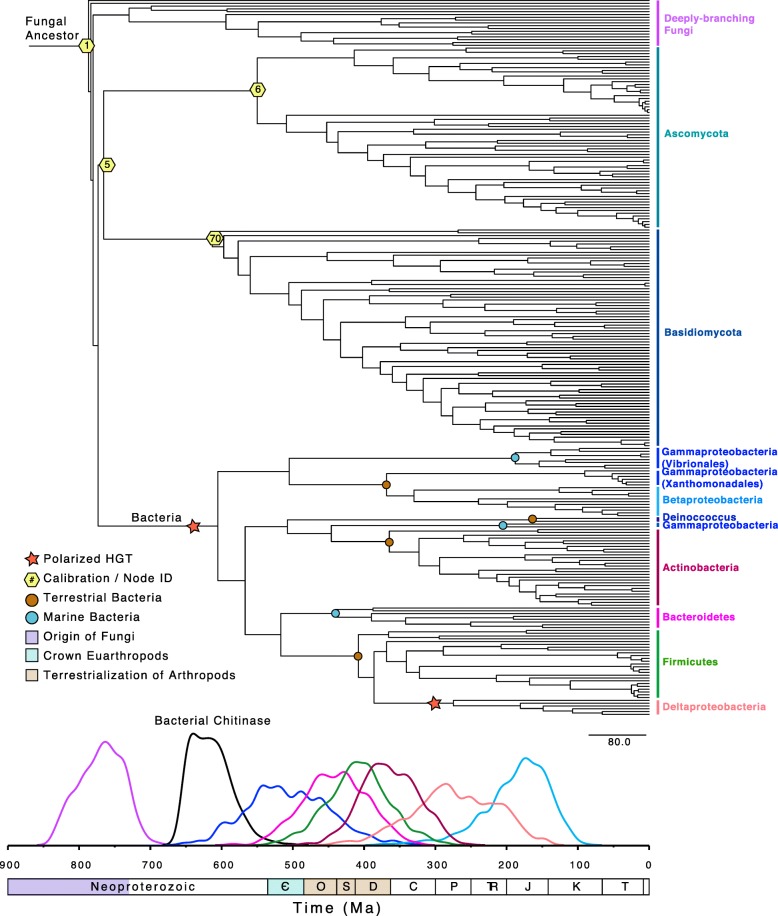
Chronogram depicting the phylogenetic relationships between chitinase homologs, and posterior age estimates obtained under the prior. This illustrates date distributions of key fungal and bacterial clades assuming a uniform distribution with uncorrelated gamma multipliers. On the bottom of the figure, the date distributions of key nodes are interwoven with events in the evolution of the major sources of environmental chitin: fungi and marine and terrestrial arthropods

The chronogram depicted in Fig. 2 shows that bacterial chitinases have a common ancestor ~ 780 Ma (Node 3, Table 3) and were acquired from Fungi prior to the evolution of marine arthropods in the Cambrian. Subsequent HGT events between bacterial groups distributed this gene, with the major bacterial clades in the tree acquiring chitinase ~ 505–188 Ma. This age range is consistent with the ecological and taxonomic dispersal of bacterial chitinases being correlated with the origin and diversification of crown group euarthropods around 521–514 Ma [29, 31]. Interestingly, four major clades of terrestrial Bacteria in the tree, Gammaproteobacteria (Xanthamonadales), Betaproteobacteria, Actinobacteria, Firmicutes, all diversify ~ 408–365 Ma, temporally consistent with the terrestrialization of arthropod groups, as terrestrial myriapods were present by 416 Ma [17, 29, 32, 33] (Fig. 1). This timing is also consistent with molecular clock evidence for the early terrestrialization of land plants (middle Cambrian – Early Ordovician) and vascular plants (Late Ordovician – Silurian) [70], and alternatively, may represent the establishment of plant-degrading Fungi in soils by 300 Ma [28].

**Table 3:** Posterior divergence time estimates. Divergence time estimates calculated under the setup described for Model 6 in Table 1. Node ID represents the node identification number as depicted in Fig. S2. Cal represents the calibration used. Posterior divergence time estimates (Mean Age) and 95% confidence intervals (CI) are reported in Ma. This table represents our preferred model assumptions and results for bacterial divergence time estimates

Node	Node ID	Cal	Mean Age	95% CI
Root	1	1145–738	787	(738–837)
Ancestral Fungi	3		780	(729–833)
Dikarya (AB Split)	5	830–518	766	(720–828)
Ascomycota	6	715–408	550	(480–618)
Basidiomycota	70	655–400	613	(566–655)
Bacteria	147		605	(537–672)
Gammaproteobacteria	148		505	(393–605)
Vibrionales	165		188	(113–278)
Betaproteobacteria	155		330	(223–442)
Bacteroidetes	174		440	(359–525)
Firmicutes	181		408	(323–498)
Deltaproteobacteria	186		275	(174–389)
Actinobacteria	215		365	(290–435)

### Testing molecular clock models

Molecular clock models as listed in Table 1 were tested to assess model parameter sensitivities. The results for Model 6 (selected for further analysis) are presented in Table 3. The results of all model outputs are listed in Additional file 4: Table S2. An analysis of these models is presented in Table 4 and further elaborated upon in the following sections. Table 4 illustrates the models excluding calibrations on Ascomycota and Basidiomycota crown groups that recover the expected age ranges for these nodes in the literature. For this analysis, Calibrations 1 and 3 were used, as these do not impose dates on crown Ascomycota or Basidiomycota clades, enabling comparison between estimated and expected model output for these clades. Table 4 shows that the 95% CI posterior ages fall within expected ranges for the uniform prior and uncorrelated gamma relaxed clock model for Ascomycota under Calibrations 1 and 3. The model ages also fall within expected ranges the uniform prior and uncorrelated gamma clock model for Basidiomycota Calibration 1; uniform prior and lognormal autocorrelated clock model for Ascomycota, Calibration 3; and birth-death prior and uniform gamma distributed model for Basidiomycota, Calibration 3. Mean ages for the birth-death prior and uncorrelated gamma model and for the uniform prior with lognormal model fall outside of expected age ranges under Calibrations 1 and 3 for Ascomycota and under Calibration 1 for Basidiomycota.

**Table 4:** Posterior date distributions for model parameters. The calibration refers to the node, model number (in parentheses), calibration age (in Ma). The node refers to either Ascomycota (A) or Basidiomycota (B). The prior refers to the tree process prior, either uniform or birth-death. The clock model refers to the relaxed clock model, either lognormal autocorrelated (LN) or uncorrelated gamma (ugam). The model ID column refers to the model number as delineated in Table 1. The posterior mean age estimates and 95% confidence intervals (CI) are in Ma as calculated in the output of each PhyloBayes model. The expected age ranges (Ma) are listed based on literature values (user priors) as noted in Table 2. The Outside Expected (OE) column lists the percent of the 95% CI outside of the expected range

Cal Node, Number, Age	Node	Prior	Clock Model	Model ID	Mean Age	95% CI		Expected		OE (%)
AB-Split (Cal 1)	Ascomycota	uniform	LN	1	651	728	580	715	408	8.8
830–518		uniform	ugam	5	559	630	481	715	408	0.0
		BD	LN	13	494	592	391	715	408	8.5
		BD	ugam	9	366	457	267	715	408	74.2
	Basidiomycota	uniform	LN	1	674	749	602	655	400	63.9
		uniform	ugam	5	628	698	559	655	400	30.9
		BD	LN	13	550	650	443	655	400	0.0
		BD	ugam	9	456	572	349	655	400	22.9
AB-Split (Cal 3)	Ascomycota	uniform	LN	3	656	731	590	715	408	11.3
830–405		uniform	ugam	7	560	637	484	715	408	0.0
		BD	LN	15	510	616	397	715	408	5.0
		BD	ugam	11	366	447	276	715	408	77.2
	Basidiomycota	uniform	LN	3	677	754	607	655	400	67.3
		uniform	ugam	7	626	687	554	655	400	24.1
		BD	LN	15	560	674	430	655	400	7.8
		BD	ugam	11	462	570	369	655	400	15.4

### Impact of the tree process prior and rate distribution model

**Fig. 3:**
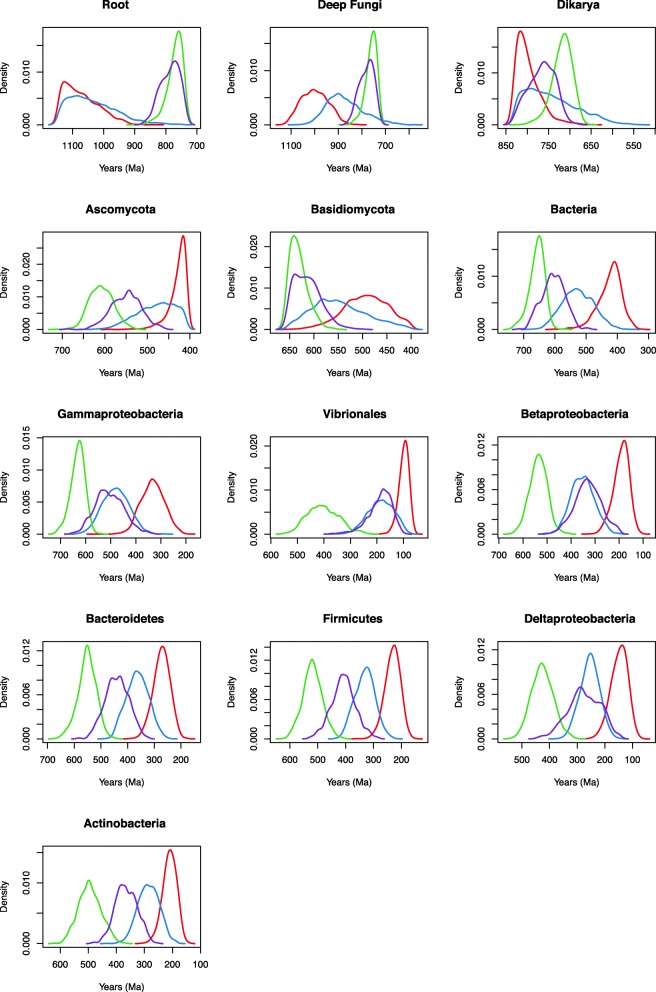
Posterior chitinase date distribution across nodes with varied model selection. Models: 10, birth-death prior and uncorrelated gamma relaxed clock model (red); 14, birth-death prior and lognormal autocorrelated relaxed clock model (blue); 2, uniform prior and lognormal autocorrelated relaxed clock model (green); and 6, and uniform prior and uncorrelated gamma relaxed clock model (purple)

The effects of the tree process prior (birth-death vs. uniform) and the rate distribution model (lognormal correlated vs. uncorrelated gamma) were evaluated (Table 4, Additional file 4: Table S2). Prior and posterior age estimates for the chitinase tree using a uniform vs. birth-death prior and lognormal vs. gamma rate distribution return different date distributions across nodes, in both bacterial and fungal groups. Across the Bacterial nodes, the uniform prior with lognormal autocorrelated clock model corresponded to the oldest date estimates across nodes, followed by the uniform prior and uncorrelated gamma model, birth-death prior and lognormal autocorrelated model, and finally the youngest birth-death prior and uncorrelated gamma relaxed clock model (Fig. 3). The birth-death prior resulted in the youngest age estimates as compared to the uniform prior (Fig. 3). The same pattern holds for the Ascomycota and Basidiomycota within the fungal nodes. However, a slightly different result is observed for the deeply-rooting fungal nodes (root, Fungi, and Dikarya). For these fungal nodes, the opposite pattern is seen with the oldest date distributions resulting from the birth-death prior and uncorrelated gamma clock model, followed by the birth-death prior and lognormal autocorrelated model, the uniform prior and uncorrelated gamma model, and finally the youngest uniform prior and lognormal autocorrelated clock model (Fig. 3). This empirical control on predicting fungal age estimates for nodes that have had their calibrations removed suggests that the uniform tree process and uncorrelated gamma rate distribution provide the most accurate age estimates for this gene family.

This model selection is also theoretically justifiable. A birth-death prior is a tree process prior that assumes a tree generated by speciation and extinction events across a lineage [49]. This assumption is violated for trees that include HGT events, especially if several such events are present. Birth-death priors are therefore not appropriate for gene trees that show histories of extensive HGT, since the underlying assumption, that nodes are distributed across a continuity of lineage speciation and extinction, is invalid. This is especially true for HGTs between microbes and eukaryotes, which sometimes have very different patterns of speciation and extinction occurring over very different timescales and sampling densities. The chitinase tree is an especially good test of these hypotheses, as in this dataset we infer multiple HGTs between Bacteria after a primary HGT from Fungi. There are many nodes that are clearly not the consequence of birth-death processes. In fact, the ecological dispersal of genes via HGT should be expected to locally increase node densities in the tree entirely independent of any underlying assumptions of speciation or extinction. In the absence of a different model sensitive to nodes mapping as transfers vs. speciation events, it is important to avoid assumptions made in the birth-death model. In addition, for many of the bacterial nodes, the uniform tree process prior results in broader prior ages than the birth-death prior. Therefore, the violation of the assumptions of a birth-death process in the bacterial chitinase tree may result in overly narrow priors that are too informative. Additionally, autocorrelated rate distribution models generally perform poorly for large evolutionary distances [46], and inspection of the gene tree does not readily reveal any lineage-specific branch length effects that suggest rate biases that would be poorly accounted for under an uncorrelated model.

Discrimination between these priors and evolutionary models would be substantially aided by the presence of crown-group calibrations within the bacterial clades recovered within the HGT recipient subtree. While diagnostic body fossils representing these microbial clades are almost certain not to be found, future studies may provide such calibrations in the form of additional HGT events, or inferred cospeciations with fossil-calibrated metazoan host lineages [71]. Such additional calibrations would also permit sensitivity analyses to be performed for the HGT-based calibrations used in this study.

### Impact of taxon sampling and fungal divergence times

The impact of taxon sampling was evaluated (Additional file 1: Table S1, Additional file 4: Table S2). Within Fungi, the chitinase gene appears to follow a history of vertical descent, and therefore better modeled under a birth-death tree process prior. Therefore, one test of the appropriateness of a birth-death process prior is if the presence of bacterial sequences within the tree impacts the effective prior ages within Fungi. Ascomycota and Basidiomycota groups each have prior ages ~ 100 Ma younger under the birth-death model when Bacteria are removed. Under the uniform model, Ascomycota is the same age whether or not Bacteria are included, while Basidiomycota is also ~ 100 Ma younger. In general, the birth-death model gives much younger prior ages, ~ 150 Ma for Bacteria and Basidiomycota, whether or not Bacteria are in the tree, and ~ 150 Ma for Ascomycota in the presence of Bacteria, and ~ 250 Ma in the absence of Bacteria. Ascomycota and Basidiomycota crown group age priors are very sensitive to the tree process prior. Therefore, we chose to use additional secondary calibrations within Dikarya to constrain the prior on the Ascomycota and Basidiomycota nodes.

### Impact of calibrations

**Fig. 4:**
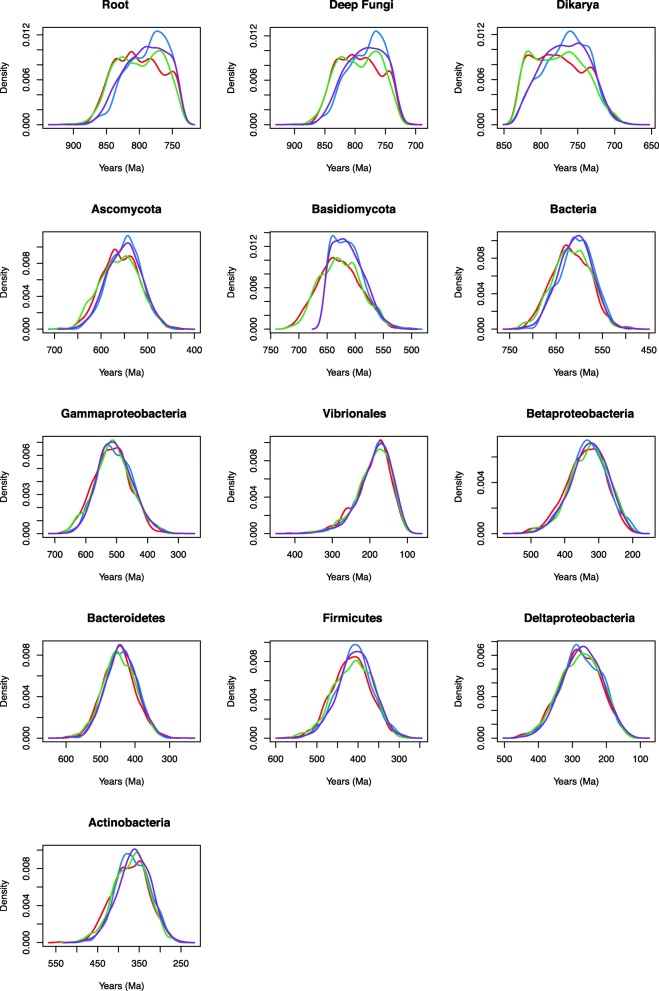
Posterior chitinase date distribution across nodes with varied calibration. Illustrates the posterior age distributions across nodes under the four calibration setups, 1 (red), 2 (blue), 3 (green) and 4 (purple). These correspond to Models 5, 6, 7, and 8

In general, the date distributions across all nodes do not appear to be very sensitive to the calibrations applied under the uniform distribution and uncorrelated gamma relaxed clock model. Because the calibrations are all roughly in the same range, it appears that all calibration results lead to similar date distributions (Fig. 4). However, Calibration 2 (calibrations on the root, split of Ascomycota and Basisiomycota, and Ascomycota and Basidiomycota crown lineages, not including the fossil minima) lead to slightly more precise peaks (Fig. 4). There are two potential problems with using single gene alignments to generate a posterior age estimate for an HGT: (1) a single gene has limited rate information from aligned sites for an informative molecular clock, and (2) if HGT increases the rate of evolution along reticulate branches due to genes evolving faster once in a recipient genome, then the posteriors will bias results towards under-estimating the ages of these groups. Therefore, we assessed whether younger posterior dates generated by the birth-death prior as compared to the uniform prior were due to the long branch separating Bacteria from Fungi in the tree. It is possible that this long branch may either be representative of a longer time interval (and thus younger crown ages) or of a faster evolutionary rate (and thus older crown ages). The maximum likelihood tree (Fig. 1 and Additional file 5: Figure S3) illustrates that when rooted, Ascomycota and Basidiomycota actually have slightly longer distances to the root, suggesting that the relative rates of evolution in this gene tree are not accelerated in the bacterial group. Consequently, the limited sequence information contained in this dataset may be used to calculate posterior age estimates that are unlikely to be biased by HGT-induced rate effects. Including additional internal constraints on the fungal clades push the priors under the uniform and birth-death models closer together for bacterial nodes. These additional secondary calibrations are thus important for constraining the tree process prior, and this type of approach may be important for using single gene HGTs to improve age estimates in general.

### Informativeness of sequence data

**Fig. 5:**
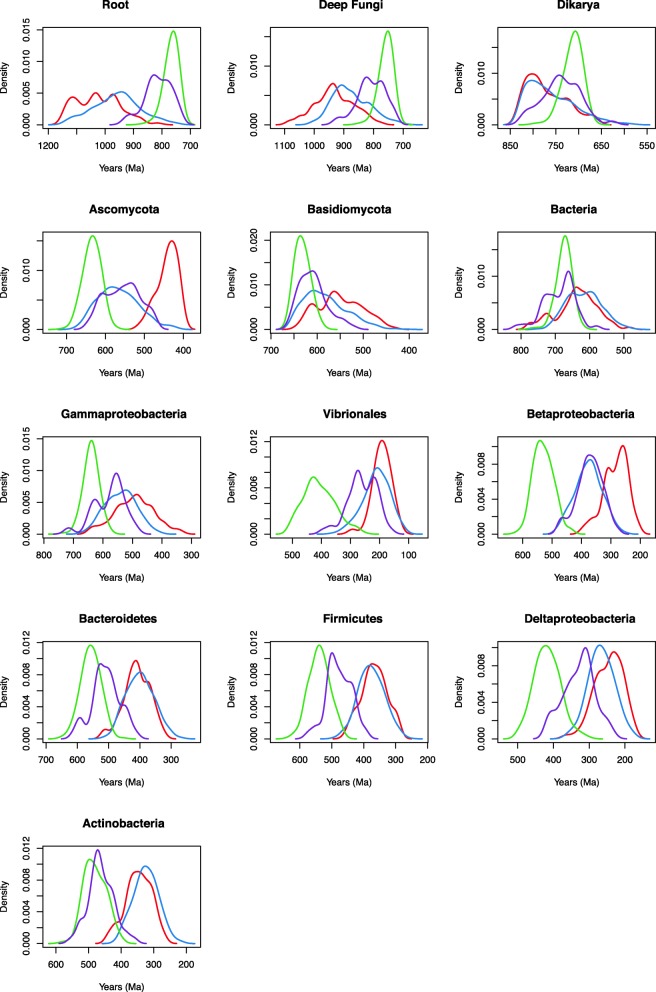
Prior date distributions across nodes. Models: 10p, birth-death prior and uncorrelated gamma relaxed clock model (red); 14p, birth-death prior and lognormal autocorrelated relaxed clock model (blue); 2p, uniform prior and lognormal autocorrelated relaxed clock model (green); and 6p, and uniform prior and uncorrelated gamma relaxed clock model (purple)

**Fig. 6:**
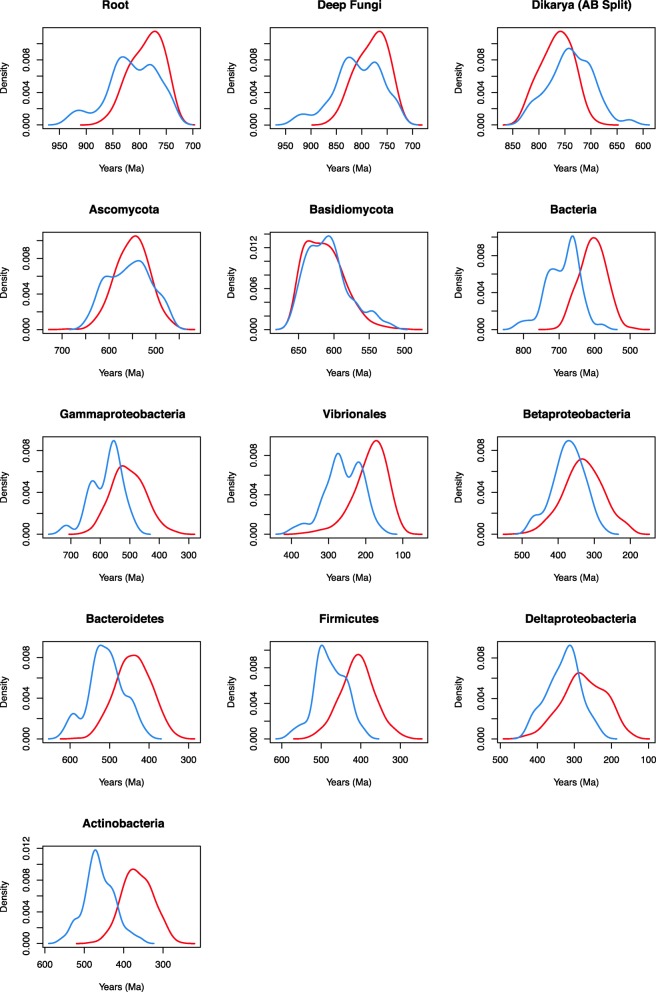
Prior vs. posterior chitinase date distributions across nodes run under the prior (blue) and including sequence data (red)

We assessed the informative of the sequence data by running PhyloBayes under the prior (effective prior, including calibrations (Additional file 6: Figure S4)). Posterior age distributions for bacterial chitinase nodes substantially differed from prior age distributions, showing that sequence data is meaningfully informing age estimates via the relaxed molecular clock (Figs. 5 and 6).

## Discussion

### Fungal origin and distribution of bacterial Chitinases

The gene tree topology for ChiC/D and its inferred rooting within Fungi show that bacterial chitinase was acquired via HGT from a fungal donor lineage. By including secondary age calibrations on nodes within Fungi, molecular clock estimates show that this gene was acquired by bacteria by 605 Ma (range of 655–566 Ma), slightly predating estimates for the evolution of crown marine euarthropods [31, 32]. While the environment of the first Fungi is uncertain, the earliest Fungi likely evolved from aquatic ancestors, and colonized land by moving from shallow marine or freshwater environments to terrestrial environments [22, 24, 72, 73]. This is consistent with the hypothesis that bacterial chitinases evolved from an aquatic ancestor. Nevertheless, the initial acquisition of bacterial chitinase is unlikely to be in response to increases in marine arthropod chitin, which is unlikely to have been widespread at that time.

The HGT between Fungi and Bacteria also seems plausible from environmental and mechanistic perspectives. Bacteria and Fungi occupy similar environments, and other bacterial chitinases within the GH18 family (e.g. ChiJ) have been hypothesized to have evolved via HGT, possibly from Fungi [37]. Following the initial transfer into a bacterial lineage, bacterial groups have all acquired chitinases from one another via subsequent HGT events, although the donors of these HGTs cannot be directly inferred from the tree topology, except in the case of Firmicutes to Deltaproteobacteria.

### Importance of chitinase evolution for dating microbial metabolisms

Several types of calibrations exist for constraining divergence time estimates for clades of organisms. Fossil node calibrations with well-defined phylogenetic histories, morphological, and age information provide some of the strongest constraints [74]. Tip-dating or Total Evidence Dating expands the utility of fossil age constraints by merging fossil species information with extant species information [51]. HGT events also constrain the relative age of donor and recipient clades [4, 5, 9]. Nearly all bacterial groups lack fossil evidence that could potentially constrain crown-group clades. There are some fossil constraints within Cyanobacteria [3, 53], and other bacterial lineages contain proxy eukaryote fossil calibrations, such as mitochondrial lineages within Alphaproteobacteria [9]. Nonetheless, major lineages such as Firmicutes, which are distant relatives to these better-calibrated groups, are difficult to date, and because they are so distant, calibrations for other regions of the tree, even if they exist, are essentially not informative. HGT-propagated calibrations are thus especially valuable for dating microbial lineages.

Substrate-specific genes, such as chitinases, are also valuable for placing absolute older-bound ages on microbial lineages, as they can be inferred to have evolved in direct response to a derived character (e.g., chitin synthesis) found within another, better-calibrated part of the Tree of Life. In the special case that these genes were acquired by multiple HGTs across diverse recipient lineages, as we observe for ChiD, a further inference can be made: HGT acquisitions in these groups are likely the result of substrate availability increasing or expanding across multiple microbial niches. This suggests that recipient clades are all of similar ages, regardless of their taxonomic diversity. In effect, the substrate-dependent dispersal of these genes act as “Standard Candles” for dating microbial groups. The concept of Standard Candles is taken from astronomy, where absolute distances of objects can be as difficult to infer as absolute dates for species divergence times. In order to help solve this problem, the known absolute luminosity of some sets of objects can be inferred from their physical properties, such as Cepheid variable stars [75] and Type IA supernovae [14]. Given their observed (apparent) luminosities, a distance calculation can then be made, and extrapolated to other objects. Similarly, substrate-dependent HGTs may permit multiple clades to be established to be within a specific absolute age range, improving divergence time estimates across the Tree of Life. Future dating efforts will likely benefit from exploring a broader set of temporally-constrained, substrate-specific HGTs. Depending upon their ubiquity, this may be a robust means of proxy dating microbial lineages, at least within the time horizon of eukaryal life for which a diagnostic fossil record exists.

Divergence time age estimates from this study can also be useful for future investigations. While a single gene, such as ChiD, contains limited sequence data for informing posterior age distributions, posterior dates from HGT-calibrated gene trees can be used as constraints that may improve accuracy in molecular clock studies using larger alignments [57, 76]. Furthermore, in at least one case, our results suggest the likely transfer of chitinase genes from within one phylogenetically distant [77] microbial clade to another: Bacilli (Firmictues) to Myxococcales (Deltaproteobacteria) (Fig. 1). Because these clades are “nested” one could therefore polarize the direction of the transfer and apply a relative age constraint between these two groups on a species tree, independent of any propagated absolute constraints. Future work should assess how the application of such constraints affect divergence time estimates on species trees containing additional sequence information and increased taxon sampling.

### Ecological implications of chitinase evolution

**Fig. 7:**
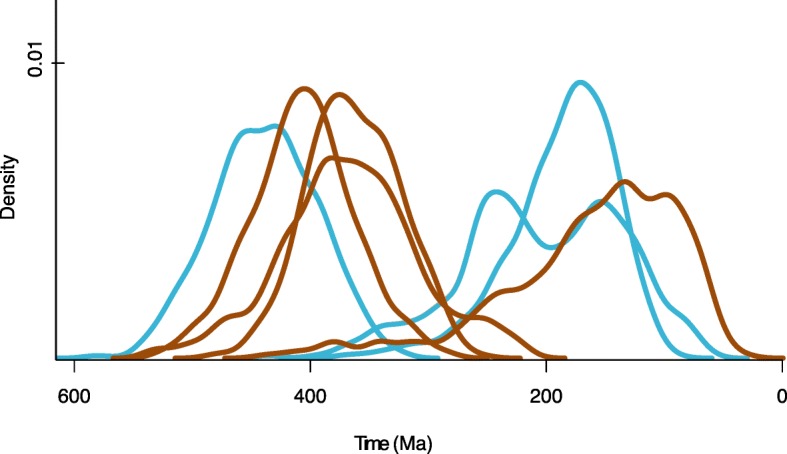
Posterior date distributions for aquatic (blue) and terrestrial (brown) bacterial chitinases. Posterior date distributions correspond to the uniform prior and uncorrelated gamma relaxed clock model with calibration 4 (Model 6). Aquatic nodes represent ancestors of groups within Vibrionales (165), Gammaproteobacteria (214), and Bacteroidetes (174). Terrestrial nodes represent ancestors of groups within Betaproteobacteria (149), Deinococcus (212), Actinobacteria (215) and Firmicutes (181)

Our results show that numerous clades of bacteria acquired chitinase genes during the early Paleozoic (Fig. 7), suggesting that their dispersal throughout the microbial world was in direct response to the evolutionary and ecological expansion of detrital-chitin producing arthropod groups. Nonetheless, it is uncertain how the primary origin of environmentally-relevant amounts of chitin has evolved through time; did this originate from fungal cell walls or detrital chitin from the molted exoskeletons of arthropods? The genomic record may aid in distinguishing these sources. Robust across our model parameters and assumptions, bacterial chitinase (ChiD) appears to have evolved from fungi, likely in response to the availability of chitin as a major structural component of fungal cell walls in the Proterozoic, prior to early arthropod evolution. Subsequent HGT and inheritance of chitinase within terrestrial bacterial clades appears to be a much more recent series of evolutionary events within the early Paleozoic, consistent with evidence for plant and arthropod terrestrialization during this time [32, 78]. The taxonomic distribution of ChiD within marine microbial groups is too sparse to infer the timing of their acquisition, or to polarize the deep HGT events between microbial lineages, which, presumably, progressed from marine to terrestrial clades (Fig. 7). The long reticulating branch leading from Fungi to the bacterial ChiD sequences suggests that the direct fungal donor clade is not represented in the current tree; this may be due to unsampled extant fungal diversity, or patterns of extinction among ancient marine fungal groups.

## Conclusion

The evolution of the metabolic degradation of detrital arthropod and fungal chitin may provide important temporal clues for dating early bacterial diversification. If bacterial chitinase genes were acquired in response to environmental chitin availability, then arthropod evolutionary history provides a prediction for the timing of these events within bacterial lineages: terrestrial bacterial chitinases should have diversified from the Cambrian into the Devonian. We independently date chitinase evolution in microbial lineages by applying molecular clocks to chitinase gene trees, constrained by fungal date calibrations tethered via HGT. We show that concordance with terrestrial arthropod evolution indeed appears to be the case, further supporting the utility of HGT-propagated fossil calibrations in accurately estimating the ages of microbial lineages.

Bacterial chitinases appear to have diversified from the time of their acquisition, roughly 600 Ma, into the last 200 Ma of Earth history (Table 3). This is consistent with the hypothesis that bacterial chitinases evolved in response to the seeding of marine and terrestrial environments with globally-significant amounts of chitin, first from fungi, then later from marine and then terrestrial arthropods. There is later evidence of at least one HGT event within bacterial lineages, from within Firmicutes to Deltaproteobacteria. Although we only assessed one chitinase gene tree in this study, future work evaluating the phylogenetic distribution of other chitinase genes will be important for quantifying chitinase evolution in marine and terrestrial environments to further test the hypothesis that the phylogenetic distribution of chitinase genes mirrors the evolution and terrestrialization of environmental chitin sources.

Further, we show the importance of prior choice, highlighting that this dataset, which includes at least one deeply-rooted HGT, violates the birth-death prior. Moreover, we argue for the use of a uniform prior, uncorrelated gamma multipliers model, and three internal secondary calibrations propagating fossil calibrations from within Fungi to Bacteria.

Finally, we suggest that our dataset does not demonstrate HGT-associated heterotachy. Thus, our fungal priors and perhaps even posterior bacterial date distributions, may be more broadly applicable for future molecular clock studies assessing the divergence times of these major clades of Bacteria.

## Additional files

Additional file 1: **Table S1.** Taxa in this study and their environment. Environment refers to the environment that these substrates operate in^1^ or where the organism was sampled from according to NCBI. Aquatic represents marine environments. Terrestrial refers to land which includes shallow freshwater ponds/swamp type sample locations. (DOCX 42 kb)

Additional file 2: **Figure S1.** PhyloBayes chitinase gene tree with posterior probabilities supporting nodes. (PDF 46 kb)

Additional file 3: **Figure S2.** Chronogram with corresponding node numbers used in analysis. Clades are annotated on corresponding nodes. (PDF 374 kb)

Additional file 4: **Table S2.** Extended Table 2 of all results and parameters tested. Prior and posterior divergence time estimates calculated under all model assumptions described in Table 1. Divergence date ranges are given in Ma. (XLSX 12 kb)

Additional file 5: **Figure S3.** Expanded RAxML gene tree with tip labels and bootstrap support values. (PDF 16 kb)

Additional file 6: **Figure S4.** Prior date distributions across nodes under the four calibration setups. Models: Calibration 1 (red), Calibration 2 (blue), Calibration 3 (green), Calibration 4 (purple). (PDF 492 kb)

## Abbreviations

BLAST: Basic Local Alignment Search Tool
Chi (A-J): Chitinase (A-J)
GH: Glycoside hydrolase
HGT: Horizontal gene transfer
Ma: Million years ago
NCBI: The National Center for Biotechnology Information
nr: Nonredundant
