# Supplementary material for: Paleozoic diversification of terrestrial chitin-degrading bacterial lineages
Source: BMC Evol Biol. 2019 Jan 28;19:34. doi: 10.1186/s12862-019-1357-8 (PMC6348609; doi:10.1186/s12862-019-1357-8)
Supplement: Supplementary file 1 — Table S1. Taxa in this study and their environment. Environment refers to the environment that these substrates operate in1 or where the organism was sampled from according to NCBI. Aquatic represents marine environments. Terrestrial refers to land which includes shallow freshwater ponds/swamp type sample locations. (DOCX 42 kb) [file 12862_2019_1357_MOESM1_ESM.docx]

**Table S1**. Taxa in this study and their environment. Environment refers to the environment that these substrates operate in^1^ or where the organism was sampled from according to NCBI. Aquatic represents marine environments. Terrestrial refers to land which includes shallow freshwater ponds/swamp type sample locations.

| **Taxon Name \| Protein ID** | **Clade** | **Taxonomy** | **Environment** |
| --- | --- | --- | --- |
| Actinobacteria_bacterium_13_2_20CM_2_71_6\|OLB77094.1 | bacteria | Actinobacteria | T |
| Actinokineospora_inagensis\|WP_026423506.1 | bacteria | Actinobacteria | T |
| Actinomadura_macra\|WP_067464737.1 | bacteria | Actinobacteria | T |
| Actinophytocola_xinjiangensis\|WP_075132815.1 | bacteria | Actinobacteria | T |
| Actinosporangium_sp_NRRL_B_3428\|WP_052581227.1 | bacteria | Actinobacteria | T |
| Actinosynnema_sp_ALI_1_44\|WP_076987693.1 | bacteria | Actinobacteria | T |
| Amycolatopsis_kentuckyensis\|WP_086844859.1 | bacteria | Actinobacteria | T |
| Asanoa_ishikariensis\|WP_090795177.1 | bacteria | Actinobacteria | T |
| Catelliglobosispora_koreensis\|WP_026207975.1 | bacteria | Actinobacteria | T |
| Cellulosimicrobium_cellulans\|WP_087471431.1 | bacteria | Actinobacteria | T |
| Glycomyces_sambucus\|WP_091045549.1 | bacteria | Actinobacteria | T |
| Herbidospora_daliensis\|WP_062439410.1 | bacteria | Actinobacteria | T |
| Kibdelosporangium_sp_MJ126_NF4\|WP_042195239.1 | bacteria | Actinobacteria | T |
| Kitasatospora_albolonga\|WP_084750020.1 | bacteria | Actinobacteria | T |
| Kribbella_sp_ALI_6_A\|WP_077015618.1 | bacteria | Actinobacteria | T |
| Lechevalieria_fradiae\|WP_090044473.1 | bacteria | Actinobacteria | T |
| Micromonospora_sp_CB01531\|WP_073839717.1 | bacteria | Actinobacteria | T |
| Microtetraspora_glauca\|WP_030497465.1 | bacteria | Actinobacteria | T |
| Mycobacterium_tuberculosis\|CNE32205.1 | bacteria | Actinobacteria | T |
| Nonomuraea_jiangxiensis\|WP_090946548.1 | bacteria | Actinobacteria | T |
| Planobispora_rosea\|WP_068921834.1 | bacteria | Actinobacteria | T |
| Planomonospora_sphaerica\|WP_068895167.1 | bacteria | Actinobacteria | T |
| Saccharothrix_sp_NRRL_B_16348\|WP_053716888.1 | bacteria | Actinobacteria | T |
| Sinosporangium_album\|WP_093169663.1 | bacteria | Actinobacteria | T |
| Streptoalloteichus_hindustanus\|WP_073483646.1 | bacteria | Actinobacteria | T |
| Streptomyces_scabrisporus\|WP_020551069.1 | bacteria | Actinobacteria | T |
| Streptosporangium_subroseum\|WP_089208112.1 | bacteria | Actinobacteria | T |
| Thermoactinospora_rubra\|WP_084964699.1 | bacteria | Actinobacteria | T |
| Aquimarina_spongiae\|SHI61232.1 | bacteria | Bacteroidetes | A |
| Chitinophaga_rupis\|WP_089906523.1 | bacteria | Bacteroidetes | T |
| Fulvivirga_imtechensis\|WP_083867410.1 | bacteria | Bacteroidetes | A |
| Microscilla_marina_ATCC_23134\|EAY30869.1 | bacteria | Bacteroidetes | A |
| Niastella_yeongjuensis\|WP_081197334.1 | bacteria | Bacteroidetes | T |
| Persicobacter_sp_JZB09\|WP_060687981.1 | bacteria | Bacteroidetes | A |
| Reichenbachiella_faecimaris\|WP_084370698.1 | bacteria | Bacteroidetes | A |
| Andreprevotia_chitinilytica\|WP_084187288.1 | bacteria | Betaproteobacteria | T |
| Chitiniphilus_shinanonensis\|WP_018748575.1 | bacteria | Betaproteobacteria | A |
| Chromobacterium_amazonense\|WP_071108352.1 | bacteria | Betaproteobacteria | A |
| Chromobacterium_haemolyticum\|WP_081574973.1 | bacteria | Betaproteobacteria | T |
| Chromobacterium_sphagni\|WP_071113284.1 | bacteria | Betaproteobacteria | T |
| Chromobacterium_violaceum\|WP_043615248.1 | bacteria | Betaproteobacteria | T |
| Mitsuaria_sp_7\|WP_082938754.1 | bacteria | Betaproteobacteria | T |
| Pelomonas_puraquae\|WP_088484787.1 | bacteria | Betaproteobacteria | T |
| Pseudogulbenkiania_ferrooxidans\|WP_031296507.1 | bacteria | Betaproteobacteria | T |
| Roseateles_depolymerans\|WP_083526021.1 | bacteria | Betaproteobacteria | T |
| Roseateles_terrae\|WP_088453654.1 | bacteria | Betaproteobacteria | T |
| Deinococcus_hopiensis_KR_140\|WP_084045460.1 | bacteria | Deinococcus | T |
| Deinococcus_maricopensis_DSM_21211\|WP_013558263.1 | bacteria | Deinococcus | T |
| Corallococcus_coralloides_DSM_2259\|WP_014399530.1 | bacteria | Deltaproteobacteria | T |
| Cystobacter_ferrugineus\|WP_084736787.1 | bacteria | Deltaproteobacteria | T |
| Melittangium_boletus_DSM_14713\|WP_095979189.1 | bacteria | Deltaproteobacteria | T |
| Myxococcus_fulvus\|WP_046715376.1 | bacteria | Deltaproteobacteria | T |
| Stigmatella_aurantiaca_DW4_3_1\|WP_013376730.1 | bacteria | Deltaproteobacteria | T |
| Anaerocolumna_xylanovorans_DSM_12503\|WP_073590321.1 | bacteria | Firmicutes | T |
| Bacillus_anthracis_str_H9401\|WP_014654726.1 | bacteria | Firmicutes | T |
| Bacillus_cereus_03BB108\|WP_001994684.1 | bacteria | Firmicutes | T |
| Bacillus_sp_FJAT_27238\|WP_016742699.1 | bacteria | Firmicutes | NR |
| Bacillus_toyonensis\|PAW47140.1 | bacteria | Firmicutes | T |
| Bacillus_wiedmannii\|WP_098079181.1 | bacteria | Firmicutes | T |
| Brevibacillus_brevis\|WP_087349255.1 | bacteria | Firmicutes | T |
| Brevibacillus_formosus\|WP_047074631.1 | bacteria | Firmicutes | T |
| Brevibacillus_sp_Leaf182\|WP_056491684.1 | bacteria | Firmicutes | T |
| Clostridium_botulinum_B_str_Osaka05\|WP_073860743.1 | bacteria | Firmicutes | T |
| Clostridium_cavendishii_DSM_21758\|SHI69443.1 | bacteria | Firmicutes | T |
| Clostridium_sp_ND2\|WP_084764479.1 | bacteria | Firmicutes | NR |
| Cohnella_sp_CIP_111063\|WP_094044771.1 | bacteria | Firmicute | T |
| Kurthia_gibsonii\|AFI72779.1 | bacteria | Firmicutes | T |
| Paenibacillus_assamensis\|WP_051217339.1 | bacteria | Firmicutes | T |
| Paenibacillus_ehimensis\|WP_025852116.1 | bacteria | Firmicutes | T |
| Paenibacillus_elgii\|WP_063184838.1 | bacteria | Firmicutes | T |
| Paenibacillus_swuensis\|WP_068603214.1 | bacteria | Firmicutes | T |
| Paenibacillus_taiwanensis\|WP_051287538.1 | bacteria | Firmicutes | T |
| Paenibacillus_tianmuensis\|WP_090673949.1 | bacteria | Firmicutes | T |
| Paenibacillus_tyrfis\|WP_036687954.1 | bacteria | Firmicutes | T |
| Paenibacillus\|WP_081717951.1 | bacteria | Firmicutes | T |
| Paludifilum_halophilum\|WP_094265818.1 | bacteria | Firmicutes | T |
| Streptococcus_pneumoniae\|CKG26627.1 | bacteria | Firmicutes | T |
| Thermoactinomyces_daqus\|WP_081944003.1 | bacteria | Firmicutes | T |
| Thermoactinomyces_vulgaris\|WP_022737757.1 | bacteria | Firmicutes | T |
| Hahella_chejuensis_KCTC_2396\|WP_011394837.1 | bacteria | Gammaproteobacteria | A |
| Aliivibrio_fischeri\|WP_065597214.1 | bacteria | Gammaproteobacteria | A |
| Aliivibrio_wodanis\|WP_061013584.1 | bacteria | Gammaproteobacteria | A |
| Cellvibrio_sp_pealriver\|WP_049631752.1 | bacteria | Gammaproteobacteria | A |
| Lysobacter_antibioticus\|WP_079248132.1 | bacteria | Gammaproteobacteria | T |
| Lysobacter_capsici\|ALN87908.1 | bacteria | Gammaproteobacteria | T |
| Lysobacter_enzymogenes\|WP_082644261.1 | bacteria | Gammaproteobacteria | T |
| Lysobacter_gummosus\|ALN93704.1 | bacteria | Gammaproteobacteria | T |
| Microbulbifer_sp_HZ11\|WP_081847999.1 | bacteria | Gammaproteobacteria | A |
| Photobacterium_jeanii\|WP_068327670.1 | bacteria | Gammaproteobacteria | A |
| Pseudoxanthomonas_sp_CF125\|SDQ75949.1 | bacteria | Gammaproteobacteria | T |
| Vibrio_cholerae\|WP_095481188.1 | bacteria | Gammaproteobacteria | A |
| Vibrio_coralliilyticus\|WP_038509898.1 | bacteria | Gammaproteobacteria | A |
| Vibrio_metoecus\|KQA22300.1 | bacteria | Gammaproteobacteria | T |
| Vibrio_nigripulchritudo\|WP_022594672.1 | bacteria | Gammaproteobacteria | A |
| Vibrio_parahaemolyticus\|WP_031855765.1 | bacteria | Gammaproteobacteria | A |
| Xanthomonas_sp_AK\|BAA36460.1 | bacteria | Gammaproteobacteria | T |
| Aschersonia_aleyrodis_RCEF_2490\|KZZ88173.1 | fungi | Ascomycota |  |
| Beauveria_bassiana_ARSEF_2860\|XP_008603412.1 | fungi | Ascomycota |  |
| Claviceps_purpurea_20_1\|CCE29524.1 | fungi | Ascomycota |  |
| Colletotrichum_gloeosporioides_Cg_14\|EQB55915.1 | fungi | Ascomycota |  |
| Colletotrichum_higginsianum_IMI_349063\|XP_018160050.1 | fungi | Ascomycota |  |
| Coniochaeta_ligniaria_NRRL_30616\|OIW32936.1 | fungi | Ascomycota |  |
| Cordyceps_brongniartii_RCEF_3172\|OAA33915.1 | fungi | Ascomycota |  |
| Cordyceps_militaris\|ATY67196.1 | fungi | Ascomycota |  |
| Diaporthe_ampelina\|KKY33732.1 | fungi | Ascomycota |  |
| Diaporthe_helianthi\|POS73161.1 | fungi | Ascomycota |  |
| Drechmeria_coniospora\|KYK61674.1 | fungi | Ascomycota |  |
| Escovopsis_weberi\|KOS21945.1 | fungi | Ascomycota |  |
| Fonsecaea_multimorphosa_CBS_102226\|XP_016628205.1 | fungi | Ascomycota |  |
| fungal_sp_No_14919\|GAW12378.1 | fungi | Ascomycota |  |
| Fusarium_avenaceum\|KIL91362.1 | fungi | Ascomycota |  |
| Fusarium_fujikuroi_IMI_58289\|XP_023429066.1 | fungi | Ascomycota |  |
| Fusarium_graminearum_PH_1\|XP_011324582.1 | fungi | Ascomycota |  |
| Fusarium_langsethiae\|KPA45507.1 | fungi | Ascomycota |  |
| Fusarium_mangiferae\|CVK87365.1 | fungi | Ascomycota |  |
| Fusarium_nygamai\|PNP84899.1 | fungi | Ascomycota |  |
| Fusarium_oxysporum_FOSC_3_a\|EWZ02462.1 | fungi | Ascomycota |  |
| Fusarium_poae\|OBS21759.1 | fungi | Ascomycota |  |
| Fusarium_proliferatum\|CVK87747.1 | fungi | Ascomycota |  |
| Fusarium_pseudograminearum_CS3096\|XP_009254403.1 | fungi | Ascomycota |  |
| Fusarium_sp_FIESC_5_CS3069\|CEG04533.1 | fungi | Ascomycota |  |
| Fusarium_verticillioides_7600\|XP_018749091.1 | fungi | Ascomycota |  |
| Gaeumannomyces_tritici_R3_111a_1\|XP_009219406.1 | fungi | Ascomycota |  |
| Hypocrella_siamensis\|ALI93553.1 | fungi | Ascomycota |  |
| Hypoxylon_sp_CI_4A\|OTB05187.1 | fungi | Ascomycota |  |
| Isaria_fumosorosea_ARSEF_2679\|XP_018702722.1 | fungi | Ascomycota |  |
| Magnaporthe_oryzae_70_15\|XP_003714897.1 | fungi | Ascomycota |  |
| Metarhizium_acridum_CQMa_102\|XP_007815036.1 | fungi | Ascomycota |  |
| Metarhizium_album_ARSEF_1941\|KHN93916.1 | fungi | Ascomycota |  |
| Metarhizium_anisopliae_BRIP_53293\|KJK76262.1 | fungi | Ascomycota |  |
| Metarhizium_brunneum_ARSEF_3297\|XP_014539677.1 | fungi | Ascomycota |  |
| Metarhizium_guizhouense_ARSEF_977\|KID83374.1 | fungi | Ascomycota |  |
| Metarhizium_majus_ARSEF_297\|XP_014573577.1 | fungi | Ascomycota |  |
| Metarhizium_rileyi_RCEF_4871\|OAA44119.1 | fungi | Ascomycota |  |
| Metarhizium_robertsii_ARSEF_23\|XP_007823947.1 | fungi | Ascomycota |  |
| Nectria_haematococca_mpVI_77_13_4\|XP_003050159.1 | fungi | Ascomycota |  |
| Neonectria_ditissima\|KPM38261.1 | fungi | Ascomycota |  |
| Neurospora_crassa_OR74A\|XP_011395327.1 | fungi | Ascomycota |  |
| Neurospora_tetrasperma_FGSC_2508\|XP_009853080.1 | fungi | Ascomycota |  |
| Ophiostoma_piceae_UAMH_11346\|EPE05896.1 | fungi | Ascomycota |  |
| Phaeoacremonium_minimum_UCRPA7\|XP_007917527.1 | fungi | Ascomycota |  |
| Phialophora_americana\|KIW68338.1 | fungi | Ascomycota |  |
| Pochonia_chlamydosporia_170\|XP_018136544.1 | fungi | Ascomycota |  |
| Podospora_anserina_S_mat_\|XP_001904165.1 | fungi | Ascomycota |  |
| Purpureocillium_lilacinum\|XP_018179211.1 | fungi | Ascomycota |  |
| Rosellinia_necatrix\|GAP83950.1 | fungi | Ascomycota |  |
| Sordaria_macrospora_k_hell\|XP_003348594.1 | fungi | Ascomycota |  |
| Sporothrix_insectorum_RCEF_264\|OAA58430.1 | fungi | Ascomycota |  |
| Stachybotrys_chartarum_IBT_40293\|KFA46477.1 | fungi | Ascomycota |  |
| Stachybotrys_chlorohalonata_IBT_40285\|KFA60985.1 | fungi | Ascomycota |  |
| Thielavia_terrestris_NRRL_8126\|XP_003654697.1 | fungi | Ascomycota |  |
| Torrubiella_hemipterigena\|CEJ94273.1 | fungi | Ascomycota |  |
| Trichoderma_atroviride_IMI_206040\|XP_013945238.1 | fungi | Ascomycota |  |
| Trichoderma_gamsii\|XP_018659094.1 | fungi | Ascomycota |  |
| Trichoderma_guizhouense\|OPB46374.1 | fungi | Ascomycota |  |
| Trichoderma_harzianum\|PNP57499.1 | fungi | Ascomycota |  |
| Trichoderma_parareesei\|OTA01922.1 | fungi | Ascomycota |  |
| Trichoderma_reesei\|CAZ16624.1 | fungi | Ascomycota |  |
| Trichoderma_virens_Gv29_8\|XP_013952675.1 | fungi | Ascomycota |  |
| Ustilaginoidea_virens\|KDB16030.1 | fungi | Ascomycota |  |
| Valsa_mali\|KUI66287.1 | fungi | Ascomycota |  |
| Agaricus_bisporus_var_burnettii_JB137_S8\|XP_007327593.1 | fungi | Basidiomycota |  |
| Amanita_thiersii_Skay4041\|PFH50181.1 | fungi | Basidiomycota |  |
| Armillaria_gallica\|PBK99462.1 | fungi | Basidiomycota |  |
| Armillaria_ostoyae\|SJL03806.1 | fungi | Basidiomycota |  |
| Armillaria_solidipes\|PBK72370.1 | fungi | Basidiomycota |  |
| Auricularia_subglabra_TFB_10046_SS5\|XP_007352083.1 | fungi | Basidiomycota |  |
| Calocera_cornea_HHB12733\|KZT52238.1 | fungi | Basidiomycota |  |
| Calocera_viscosa_TUFC12733\|KZP01089.1 | fungi | Basidiomycota |  |
| Coniophora_puteana_RWD_64_598_SS2\|XP_007770490.1 | fungi | Basidiomycota |  |
| Cryptococcus_depauperatus_CBS_7841\|ODN91132.1 | fungi | Basidiomycota |  |
| Cryptococcus_gattii_E566\|KIY34695.1 | fungi | Basidiomycota |  |
| Cryptococcus_neoformans_var_grubii\|OWZ72202.1 | fungi | Basidiomycota |  |
| Cylindrobasidium_torrendii_FP15055_ss_10\|KIY62608.1 | fungi | Basidiomycota |  |
| Daedalea_quercina_L_15889\|KZT71956.1 | fungi | Basidiomycota |  |
| Dichomitus_squalens_LYAD_421_SS1\|XP_007362933.1 | fungi | Basidiomycota |  |
| Exidia_glandulosa_HHB12029\|KZV81950.1 | fungi | Basidiomycota |  |
| Fibroporia_radiculosa\|XP_012181105.1 | fungi | Basidiomycota |  |
| Fibularhizoctonia_sp_CBS_109695\|KZP14692.1 | fungi | Basidiomycota |  |
| Fomitiporia_mediterranea_MF3_22\|XP_007261858.1 | fungi | Basidiomycota |  |
| Fomitopsis_pinicola_FP_58527_SS1\|EPT05438.1 | fungi | Basidiomycota |  |
| Galerina_marginata_CBS_339_88\|KDR80726.1 | fungi | Basidiomycota |  |
| Ganoderma_sinense_ZZ0214_1\|PIL36523.1 | fungi | Basidiomycota |  |
| Gelatoporia_subvermispora_B\|EMD33176.1 | fungi | Basidiomycota |  |
| Gloeophyllum_trabeum_ATCC_11539\|XP_007865094.1 | fungi | Basidiomycota |  |
| Grifola_frondosa\|OBZ73901.1 | fungi | Basidiomycota |  |
| Gymnopus_luxurians_FD_317_M1\|KIK68276.1 | fungi | Basidiomycota |  |
| Heterobasidion_irregulare_TC_32_1\|XP_009549140.1 | fungi | Basidiomycota |  |
| Hydnomerulius_pinastri_MD_312\|KIJ57853.1 | fungi | Basidiomycota |  |
| Hypholoma_sublateritium_FD_334_SS_4\|KJA28076.1 | fungi | Basidiomycota |  |
| Jaapia_argillacea_MUCL_33604\|KDQ61715.1 | fungi | Basidiomycota |  |
| Kockovaella_imperatae\|XP_021871935.1 | fungi | Basidiomycota |  |
| Kwoniella_dejecticola_CBS_10117\|XP_018260439.1 | fungi | Basidiomycota |  |
| Kwoniella_heveanensis_CBS_569\|OCF46041.1 | fungi | Basidiomycota |  |
| Kwoniella_mangroviensis_CBS_8886\|XP_019003580.1 | fungi | Basidiomycota |  |
| Kwoniella_pini_CBS_10737\|XP_019009183.1 | fungi | Basidiomycota |  |
| Laetiporus_sulphureus_93_53\|KZT09935.1 | fungi | Basidiomycota |  |
| Lentinula_edodes\|GAW00447.1 | fungi | Basidiomycota |  |
| Leucoagaricus_sp_SymC_cos\|KXN83991.1 | fungi | Basidiomycota |  |
| Melanopsichium_pennsylvanicum_4\|CDI53624.1 | fungi | Basidiomycota |  |
| Moesziomyces_antarcticus\|XP_014657252.1 | fungi | Basidiomycota |  |
| Moniliophthora_roreri_MCA_2997\|XP_007844272.1 | fungi | Basidiomycota |  |
| Naematelia_encephala\|ORY26060.1 | fungi | Basidiomycota |  |
| Neolentinus_lepideus_HHB14362_ss_1\|KZT18638.1 | fungi | Basidiomycota |  |
| Obba_rivulosa\|OCH85900.1 | fungi | Basidiomycota |  |
| Paxillus_involutus_ATCC_200175\|KIJ20918.1 | fungi | Basidiomycota |  |
| Peniophora_sp_CONT\|KZV61339.1 | fungi | Basidiomycota |  |
| Phanerochaete_carnosa_HHB_10118_sp\|XP_007400730.1 | fungi | Basidiomycota |  |
| Phellinus_noxius\|PAV18116.1 | fungi | Basidiomycota |  |
| Phlebia_centrifuga\|OKY58257.1 | fungi | Basidiomycota |  |
| Phlebiopsis_gigantea_11061_1_CR5_6\|KIP08831.1 | fungi | Basidiomycota |  |
| Piloderma_croceum_F_1598\|KIM83828.1 | fungi | Basidiomycota |  |
| Pisolithus_microcarpus_441\|KIK23049.1 | fungi | Basidiomycota |  |
| Pisolithus_tinctorius_Marx_270\|KIO10841.1 | fungi | Basidiomycota |  |
| Plicaturopsis_crispa_FD_325_SS_3\|KII93375.1 | fungi | Basidiomycota |  |
| Postia_placenta_Mad_698_R\|XP_002475036.1 | fungi | Basidiomycota |  |
| Pseudozyma_hubeiensis_SY62\|XP_012187014.1 | fungi | Basidiomycota |  |
| Punctularia_strigosozonata_HHB_11173_SS5\|XP_007386461.1 | fungi | Basidiomycota |  |
| Pycnoporus_coccineus_BRFM310\|OSD02937.1 | fungi | Basidiomycota |  |
| Rhizopogon_vinicolor_AM_OR11_026\|OAX37953.1 | fungi | Basidiomycota |  |
| Sanghuangporus_baumii\|OCB91529.1 | fungi | Basidiomycota |  |
| Schizopora_paradoxa\|KLO18244.1 | fungi | Basidiomycota |  |
| Scleroderma_citrinum_Foug_A\|KIM58986.1 | fungi | Basidiomycota |  |
| Serpula_lacrymans_var_lacrymans_S7_9\|XP_007320926.1 | fungi | Basidiomycota |  |
| Sistotremastrum_niveocremeum_HHB9708\|KZS88861.1 | fungi | Basidiomycota |  |
| Sistotremastrum_suecicum_HHB10207_ss_3\|KZT36940.1 | fungi | Basidiomycota |  |
| Sporisorium_reilianum_f_sp_reilianum\|SJX62990.1 | fungi | Basidiomycota |  |
| Stereum_hirsutum_FP_91666_SS1\|XP_007304512.1 | fungi | Basidiomycota |  |
| Suillus_luteus_UH_Slu_Lm8_n1\|KIK48287.1 | fungi | Basidiomycota |  |
| Termitomyces_sp_J132\|KNZ74502.1 | fungi | Basidiomycota |  |
| Trametes_cinnabarina\|CDO78089.1 | fungi | Basidiomycota |  |
| Trametes_pubescens\|OJT02884.1 | fungi | Basidiomycota |  |
| Trametes_versicolor_FP_101664_SS1\|XP_008038395.1 | fungi | Basidiomycota |  |
| Tremella_mesenterica_DSM_1558\|XP_007003148.1 | fungi | Basidiomycota |  |
| Tsuchiyaea_wingfieldii_CBS_7118\|XP_019032869.1 | fungi | Basidiomycota |  |
| Ustilago_bromivora\|SAM82108.1 | fungi | Basidiomycota |  |
| Wolfiporia_cocos_MD_104_SS10\|PCH44331.1 | fungi | Basidiomycota |  |
| Basidiobolus_meristosporus_CBS_931_73\|ORX91522.1 | fungi | Basidiomycota |  |
| Hesseltinella_vesiculosa\|ORX58941.1 | fungi | Basidiomycota |  |
| Allomyces_macrogynus_ATCC_38327\|KNE60875.1 | fungi | Blastocladiomycota |  |
| Catenaria_anguillulae_PL171\|ORZ38929.1 | fungi | Blastocladiomycota |  |
| Gonapodya_prolifera_JEL478\|KXS16685.1 | fungi | Chytridiomycota |  |
| Rhizoclosmatium_globosum\|ORY41636.1 | fungi | Chytridiomycota |  |
| Spizellomyces_punctatus_DAOM_BR117\|XP_016608094.1 | fungi | Chytridiomycota |  |
| Rozella_allomycis_CSF55\|EPZ32890.1 | fungi | Cryptomycota |  |
| Absidia_repens\|ORZ23163.1 | fungi | Mucoromycota |  |
| Bifiguratus_adelaidae\|OZJ02284.1 | fungi | Mucoromycota |  |
| Lichtheimia_corymbifera_JMRC_FSU_9682\|CDH53810.1 | fungi | Mucoromycota |  |
| Mucor_ambiguus\|GAN00716.1 | fungi | Mucoromycota |  |
| Phycomyces_blakesleeanus_NRRL_1555__\|XP_018290979.1 | fungi | Mucoromycota |  |
| Rhizopus_microsporus_ATCC_52813\|XP_023471300.1 | fungi | Mucoromycota |  |
| Syncephalastrum_racemosum\|ORY99444.1 | fungi | Mucoromycota |  |
| Coemansia_reversa_NRRL_1564\|PIA17930.1 | fungi | Zoopagomycota |  |
| Conidiobolus_coronatus_NRRL_28638\|KXN70579.1 | fungi | Zoopagomycota |  |
| Linderina_pennispora\|ORX67140.1 | fungi | Zoopagomycota |  |
| Pandora_neoaphidis\|APU66165.1 | fungi | Zoopagomycota |  |
