# Supplementary figures and images for: Paleozoic diversification of terrestrial chitin-degrading bacterial lineages
Source: BMC Evol Biol. 2019 Jan 28;19:34. doi: 10.1186/s12862-019-1357-8 (PMC6348609; doi:10.1186/s12862-019-1357-8)

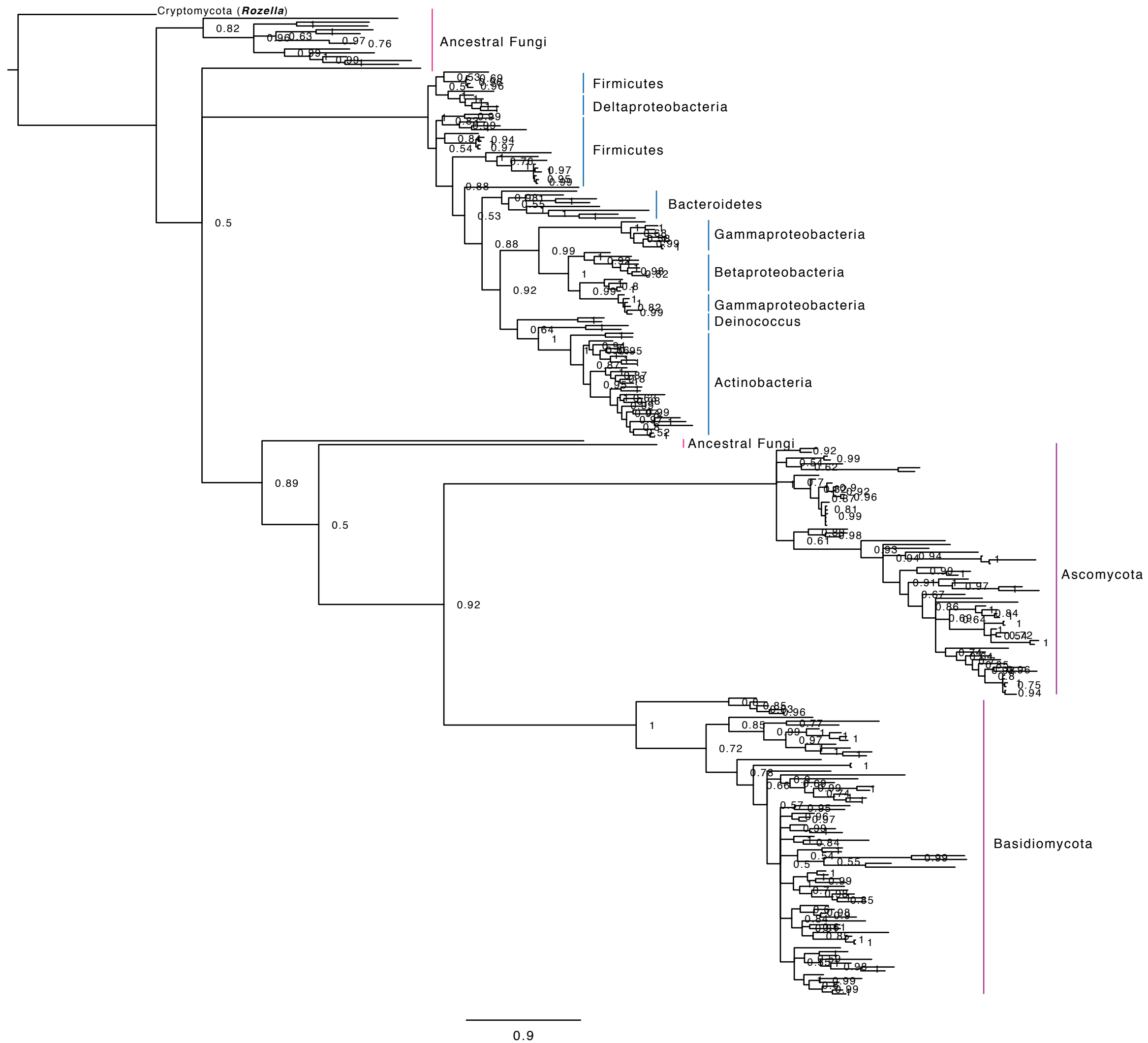

Supplement: Supplementary file 2 — Figure S1. PhyloBayes chitinase gene tree with posterior probabilities supporting nodes. (PDF 46 kb) [file 12862_2019_1357_MOESM2_ESM.pdf]

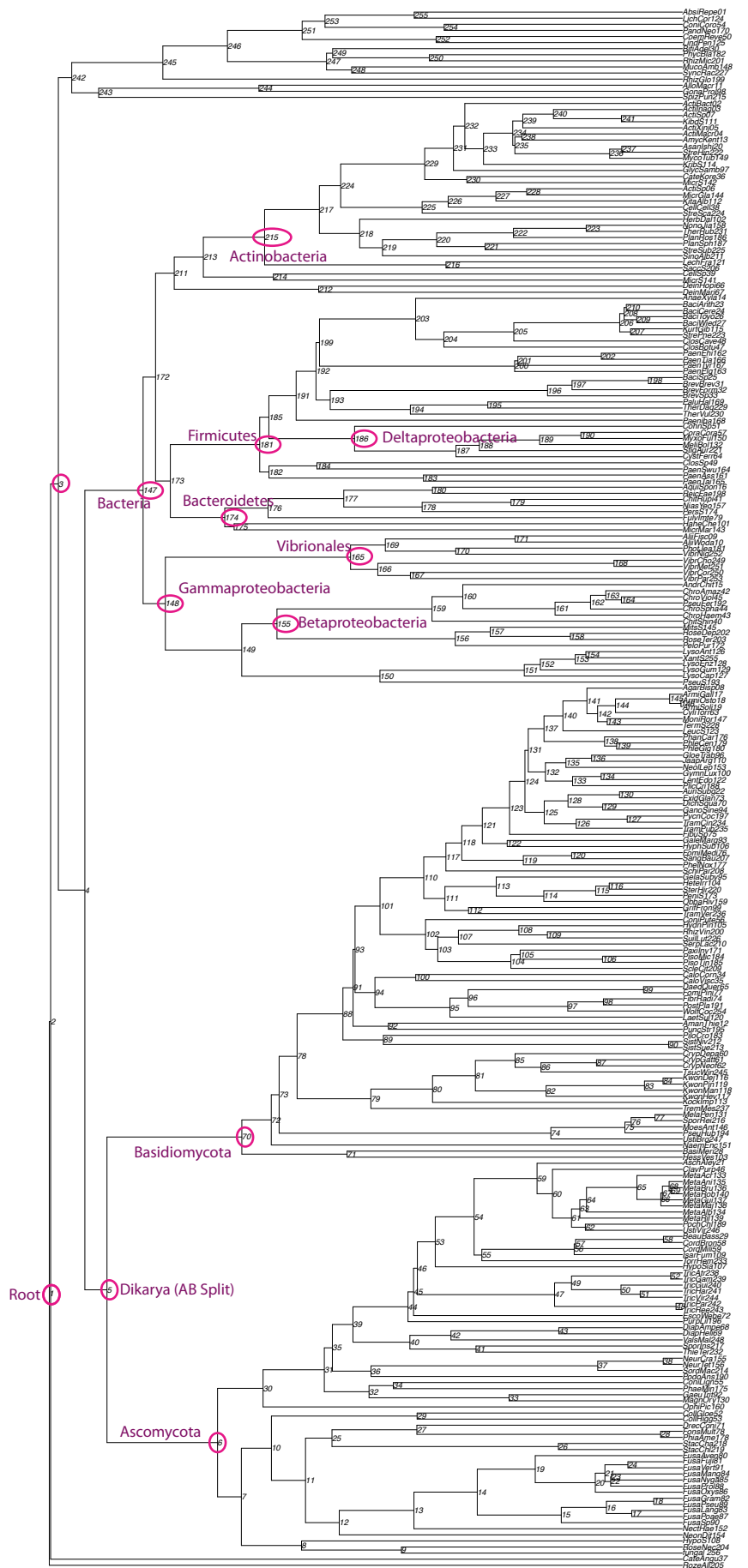

Supplement: Supplementary file 3 — Figure S2. Chronogram with corresponding node numbers used in analysis. Clades are annotated on corresponding nodes. (PDF 374 kb) [file 12862_2019_1357_MOESM3_ESM.pdf]

RozeAll205

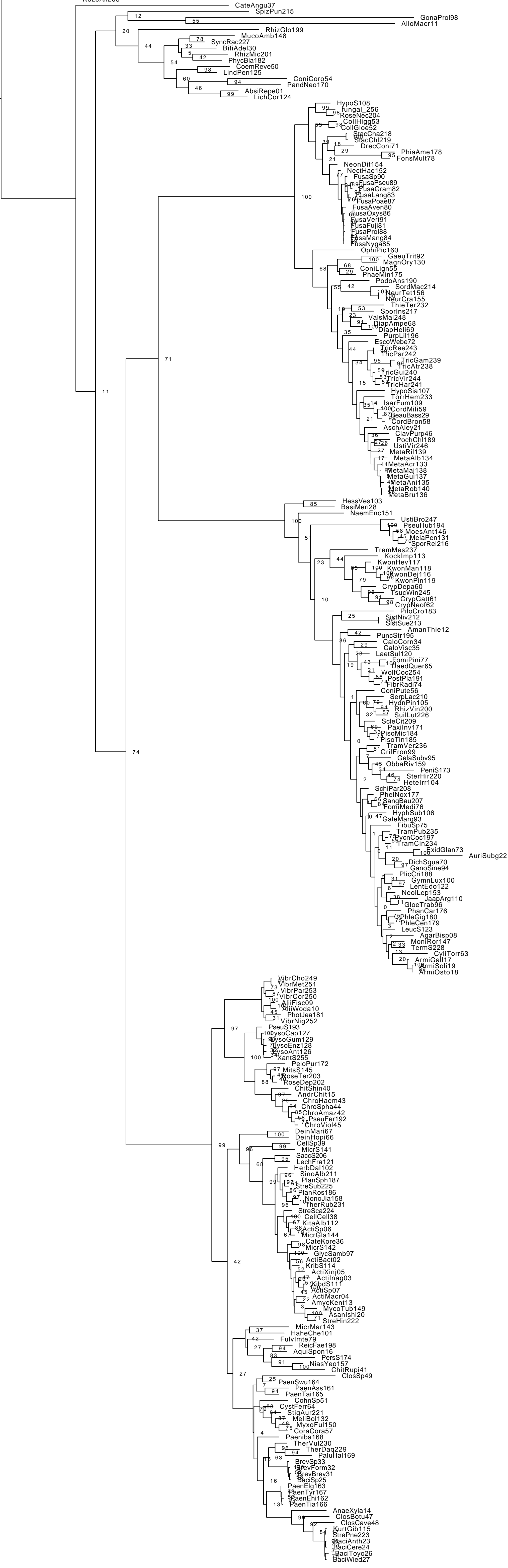

Supplement: Supplementary file 5 — Figure S3. Expanded RAxML gene tree with tip labels and bootstrap support values. (PDF 16 kb) [file 12862_2019_1357_MOESM5_ESM.pdf]
