# Supplementary material for: Paleozoic diversification of terrestrial chitin-degrading bacterial lineages
Source: BMC Evol Biol. 2019 Jan 28;19:34. doi: 10.1186/s12862-019-1357-8 (PMC6348609; doi:10.1186/s12862-019-1357-8)
Supplement: Supplementary file 6 — Figure S4. Prior date distributions across nodes under the four calibration setups. Models: Calibration 1 (red), Calibration 2 (blue), Calibration 3 (green), Calibration 4 (purple). (PDF 492 kb) [file 12862_2019_1357_MOESM6_ESM.pdf]

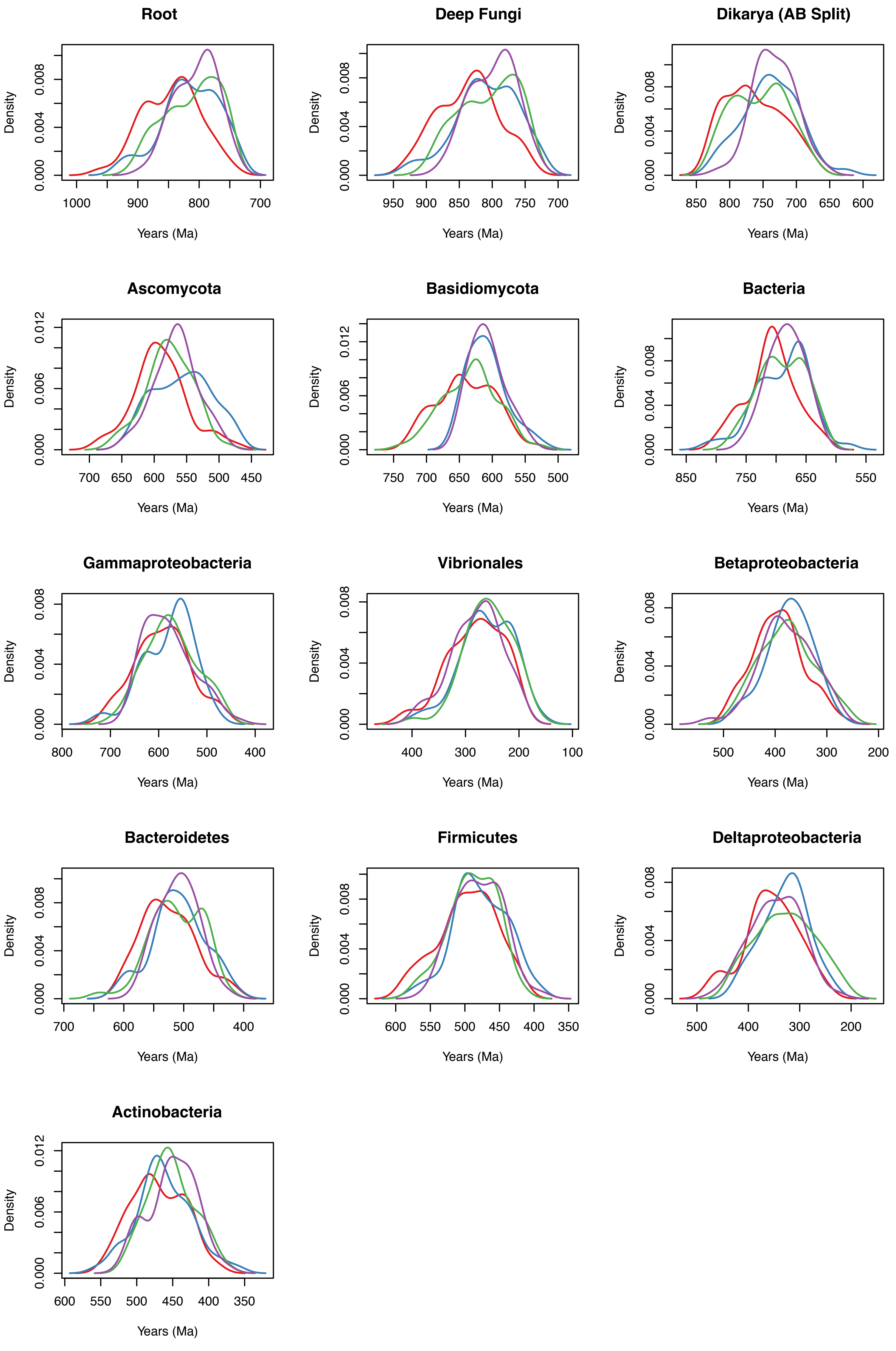
Root
Density
0.000
0.004
0.008
1000
900
800
700
Years (Ma)
Deep Fungi
Density
0.000
0.004
0.008
950
900
850
800
750
700
Years (Ma)
Dikarya (AB Split)
Density
0.000
0.004
0.008
850
800
750
700
650
600
Years (Ma)
Ascomycota
Density
0.000
0.006
0.012
700
650
600
550
500
450
Years (Ma)
Basidiomycota
Density
0.000
0.006
0.012
750
700
650
600
550
500
Years (Ma)
Bacteria
Density
0.000
0.004
0.008
850
750
650
550
Years (Ma)
Gammaproteobacteria
Density
0.000
0.004
0.008
800
700
600
500
400
Years (Ma)
Vibrionales
Density
0.000
0.004
0.008
400
300
200
100
Years (Ma)
Betaproteobacteria
Density
0.000
0.004
0.008
500
400
300
200
Years (Ma)
Bacteroidetes
Density
0.000
0.004
0.008
700
600
500
400
Years (Ma)
Firmicutes
Density
0.000
0.004
0.008
600
550
500
450
400
350
Years (Ma)
Deltaproteobacteria
Density
0.000
0.004
0.008
500
400
300
200
Years (Ma)
Actinobacteria
Density
0.000
0.006
0.012
600
550
500
450
400
350
Years (Ma)
